# An Analysis of Public Perception and Concern Toward Electronic Cigarettes: Exploring Attitudes and Profiles

**DOI:** 10.7759/cureus.47983

**Published:** 2023-10-30

**Authors:** Lisha Jiang, Shanzun Wei, Alen Sam Saji, Jue Li, Guowei Che

**Affiliations:** 1 Department of Oncology, Day Surgery Center, West China Hospital, Sichuan University, Chengdu, CHN; 2 Urology, The Second Affiliated Hospital of Nanchang University, Nanchang, CHN; 3 Department of Internal Medicine, Day Surgery Center, West China Medical School of Sichuan University, Chengdu, CHN; 4 Department of Thoracic Surgery, Lung Cancer Center, West China Medical School of Sichuan University, Chengdu, CHN; 5 Department of Thoracic Surgery, Lung Cancer Center, West China Hospital, Sichuan University, Chengdu, CHN

**Keywords:** e-smoking, vaping, e-cigarettes, electronic cigarettes, digital health tools, smoking tobacco, lung cancer, lung cancer prevention

## Abstract

Introduction

The emergence of electronic cigarettes (e-cigarettes) poses a new challenge to tobacco control efforts. With their increasing popularity, particularly among youth, public concerns have been raised in Mainland China. Further investigation is necessary to fully understand the safety and potential adverse effects of e-cigarettes.

Methods

The Baidu search index (BSI) was employed using e-cigarette related terms from January 1, 2011, to April 4, 2022. The search volume for each term was recorded and analyzed for the search trend module, geodemographic module, search-demand module, regional preferences, demographic preferences, and user demand.

Results

According to our analysis, the total BSI for the 18 e-cigarette related search keywords was 39,027,819. The average annual percentage change of BSI indicated an upward trend for each of these categories, including health issues (*p* < 0.05), definition (*p *< 0.05), product and promotions (*p *< 0.05), and policy and regulations. Of all inquiries, 59.38% originated from females and 40.62% from males. The total valid BSI for e-cigarette related words was 165,076,588, and 11.59% of all search inquiries were from individuals aged 19 years and younger. Our analysis also revealed that the public's primary concerns regarding e-cigarettes were related to their quality and potential health issues.

Conclusions

E-cigarettes enjoy great popularity nationwide, but product quality and safety are major public concerns. Regulation of e-cigarettes for their standard production, quality control, advertisement, and target customers should be implemented promptly, and the public needs to have a clear perception of e-cigarettes, especially adolescents. E-cigarette related health damages or consequences require further investigation, and advertisements and promotions for e-cigarettes should be strictly controlled by the government.

## Introduction

Tobacco use is a leading cause of preventable death [1,2], accounting for 8 million annual deaths worldwide and contributing to various non-communicable diseases (NCDs) such as cancer, heart disease, lung disease, and diabetes [3-6]. China has a significant smoking population, with more than two-fifths of the world's cigarettes consumed in the country [7,8]. The prevalence of smoking among juveniles is also a concern [9]. In addition to traditional cigarettes, electronic nicotine delivery systems (ENDS) or electronic cigarettes (e-cigarettes) have gained popularity, particularly among young people [10]. The internet has played a significant role in the widespread adoption of e-cigarettes [11,12], driven by the perception that they are less harmful than traditional cigarettes. However, the increasing use of e-cigarettes has raised public concerns about safety and adverse effects, posing a new challenge to tobacco control efforts [13-15].

The consumption of e-cigarettes is increasing in China due to the perception of providing entertainment, spiritual satisfaction, and facilitating social networking. Its popularity has grown rapidly since its launch in the commercial market, even among non-smokers [16]. Advocates claim that e-cigarettes are less hazardous than traditional tobacco and more effective for smoking cessation [17]. However, the frequency of e-cigarette use may be higher than tobacco smoking due to the addition of fragrant liquid agents and influential advertisement and promotion, which may seem harmless [18]. Currently, there is no evidence that e-cigarettes are a better alternative for smoking cessation therapy or an officially approved therapeutic device [19]. The expanded e-cigarette market in China has raised public health concerns, including issues such as youth smoking, addictive nicotine use, and smoke exposure (both active and passive) [20]. Research has shown that smoke exposure is a risk factor for various NCDs and cancers, particularly lung cancer, oropharynx cancer, and renal cancer [21]. E-cigarettes are another form of nicotine delivery, similar to cigars, pipes, water pipes, and bidis [10,22]. Nicotine inhalation can activate the sympathoadrenal system, potentially leading to cancer-related effects similar to those caused by traditional tobacco cigarette smoking [23,24]. Additionally, nicotine is an addictive substance. Given the increasing number of smokers and rates of secondhand smoke exposure in China, further investigations and reevaluation of public concerns about e-cigarettes are essential.

With the rapid development and application of information technology in healthcare, medical information has been digitized and stored in computer systems [25]. Medical informatics, also known as clinical informatics, has emerged as a discipline and has become increasingly integrated into clinical practice in recent years [26,27]. However, obtaining public perceptions and concerns within the limited time of medical consultations can be challenging. Website-based search data can help bridge this gap by analyzing user demand and public perception. Google Trends is a well-recognized academic approach for investigating public needs [28]. In this study, we used the Baidu Index, a data analysis platform based on Baidu's extensive network behavior data, to investigate infodemiology evidence of e-cigarettes, as Google is inaccessible in mainland China [29,30]. This approach allows us to gain insight into public attention toward e-cigarettes, which can potentially inform clinicians in their efforts toward lung cancer prevention and treatment, considering that lung cancer is a preventable disease in theory.

## Materials and methods

Keyword selecting and data retrieving

This study was mainly based on the temporal search trends of e-cigarette related Chinese keywords. According to the primary form of such terms available on the Baidu platform, the most common e-cigarettes-related terms consisted of “ electronic cigarettes” and “e-cigarettes ”; hence, the 18 available search keywords were identified and selected as previously described. In the current research, three typical modules including the search trend module, the geodemographic module, and the search-demand module were available for overtime search trend, regional and demographic preference, and user demand analysis [31]. Therefore, the Baidu search index (BSI) values for each e-cigarette search keyword were collected from January 1, 2011, to April 4, 2022 [32]. For the demographic portrait module and search-demand module, only 1-year data were available for retrieval; hence, data from these modules were collected from January 1, 2021, to April 4, 2022.

Data analysis

For each e-cigarette search keyword, the trend of public attention was described as the sequentially plotted daily BSI data. The search trend was described annually with the average annual percentage change (AAPC) model from the Joinpoint Regression model calculation [31]. A p-value of <0.05 was considered statistically significant. The user demand related keywords were reviewed and categorized by two investigators independently. In the event of a discrepancy, a consensus with a third investigator arbitrated the disagreement. Irrelevant inquiries were excluded.

Statistical analysis

All database was constructed using Microsoft Excel 2019 (Microsoft Corp., Redmond, WA). We used Prism 8 for macOS (version 8.4.0 (455), GraphPad Software, San Diego, CA) and SEER*Stat software, program version 4.7.0.0 (Statistical Research and Applications Branch, National Cancer Institute, Bethesda, MD) to conduct statistical analysis and create figures.

## Results

Online search trends in e-cigarettes

**Figure 1:**
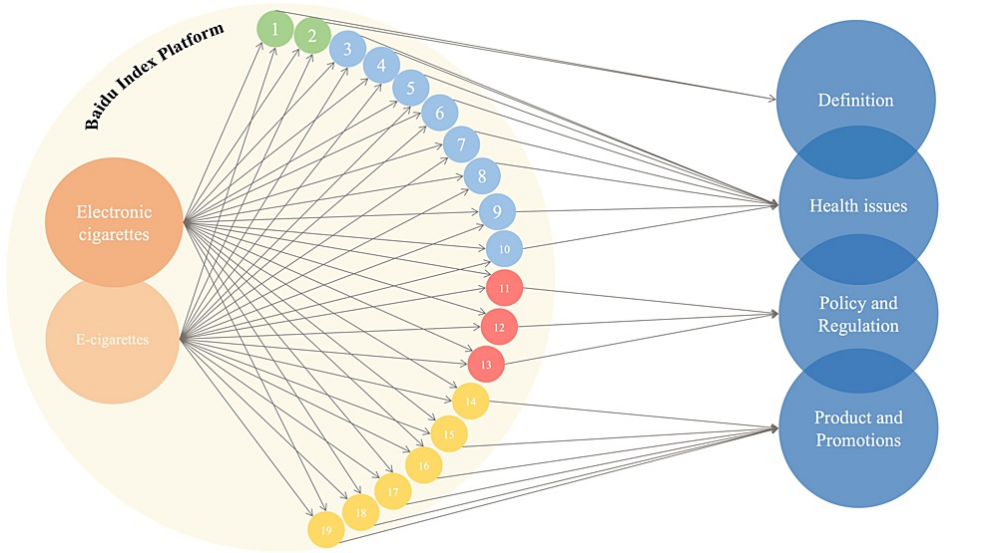
Search keywords' screening, selection, and categorization.

**Figure 2:**
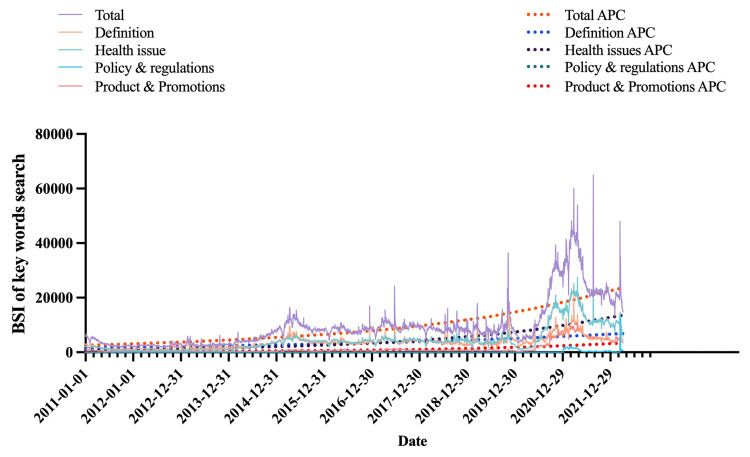
Search popularity in each e-cigarette category and the trend of each category.

We collected and summarized the total BSI of e-cigarette keywords from January 1, 2011, to April 4, 2022. According to the connotation, the retrieved 18 search keywords were itemized into four categories: 1) health issues, 2) definition, 3) policy and regulations, and 4) product and promotions (Figure 1). The total BSI was 39,027,819 during the research period. Mostly, the focus topics maintain an upward trend and rocketed dramatically in 2020. Based on the AAPC of BSI for each e-cigarette related theme, the search trends for definition (APC 13.40%, p < 0.05), health issues (APC 29.10%, p < 0.05), product and promotions (APC 29.30%, p < 0.05), and policy and regulations showed an upward trend. Additionally, the trends for each health issue and definition were found to be positively correlated and gradually escalating, while those for products and promotions and for policy and regulations remained distinct since July 5, 2020. Trends of products and promotions and of policy and regulations remained fluctuant at a relatively low range. The search volumes for all terms related to e-cigarettes spiked in 2021. The daily keywords and total inquiry times were documented, and an annual trend with the AAPC was presented, as demonstrated in Figure 2.

Geodemographic distribution differences

**Figure 3:**
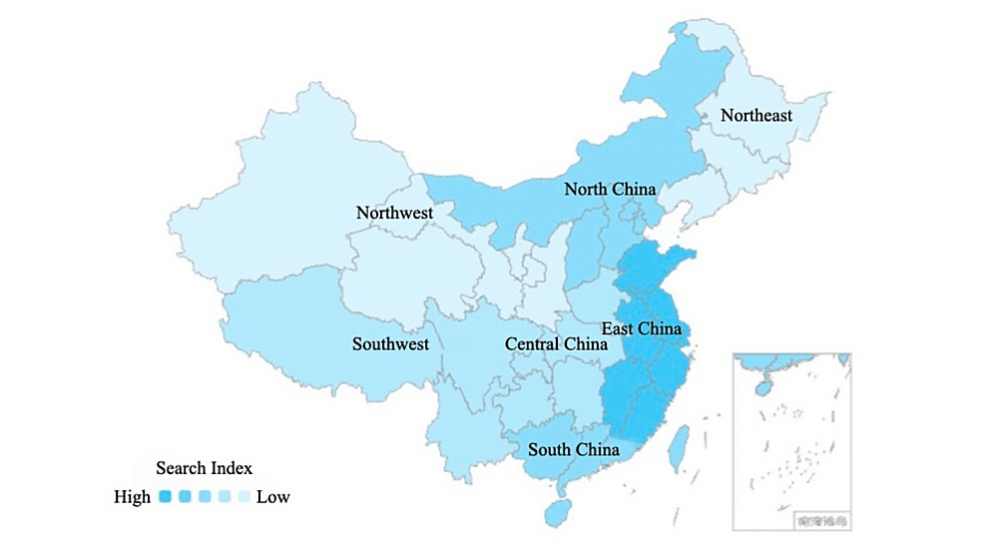
Geographical regional distribution of keywords from the official Baidu Index map.

**Figure 4:**
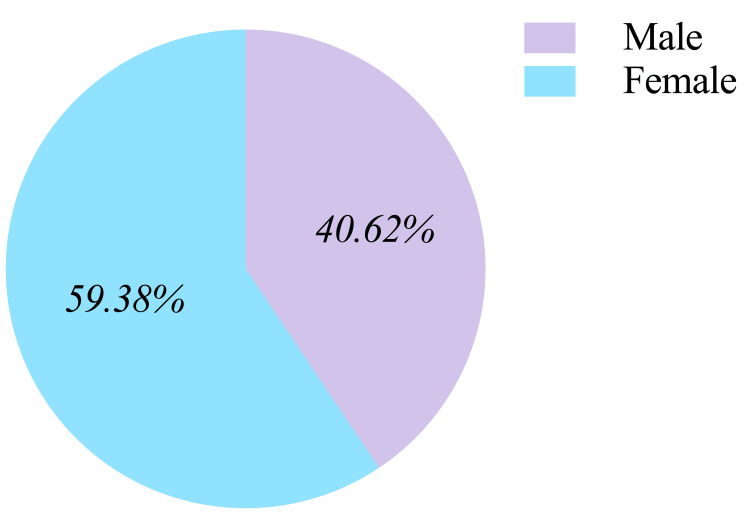
Gender distribution of e-cigarette related search.

**Figure 5:**
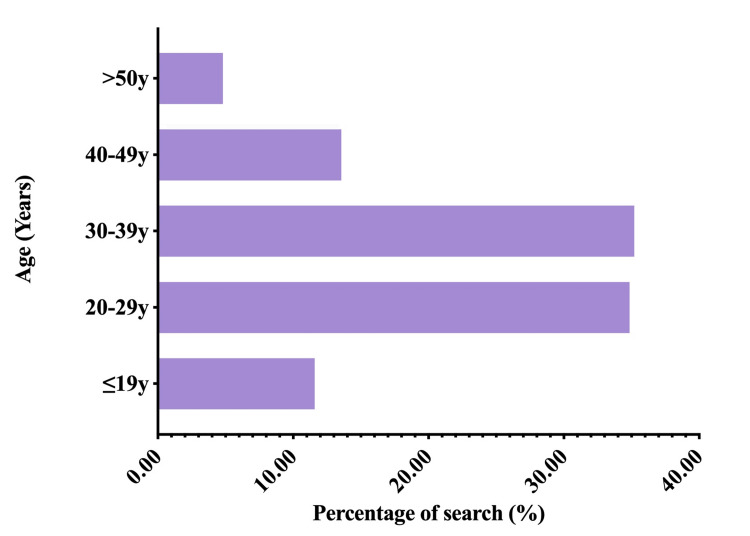
Age distribution of e-cigarette related search.

We compared geodemographic differences in searches related to the topic based on provincial data; seven geographical regions were identified according to the principle of regional division, which are northeast, north, east, south, southwest, northwest, and central China [33]. The regional geographic data were demonstrated by the official map released on the Baidu Index website [34], with the higher search index represented by a darker cyan on the map (Figure 3). East, North, and South China ranked as the top three geographical regions and took more than 60% of the search topic, accounting for 29.64%, 16.51%, and 14.11% respectively. The remaining regions, including Southwest, Central, Northwest, and Northeast China, had a relatively even distribution of searches, accounting for 11.53%, 11.17%, 8.81%, and 8.23%, respectively. Demographic distributions also revealed differences by gender; 59.38% of all inquiries were recorded from females, which was 18.76% higher than males (Figure 4). Regarding the age demographics, ages between 20 and 39 years accounted for more than 70% of the searches (34.85% from ages between 20 and 29 years and 35.20% from ages between 30 and 39 years), ages between 40 and 49 years accounted for 13.55% of the searches, and those aged over 50 years accounted only for 4.8% of the searches. Furthermore, it should be noted that those aged 19 years old and under accounted for 11.59% of all searches (Figure 5).

E-cigarette related search keywords and search frequency

**Figure 6:**
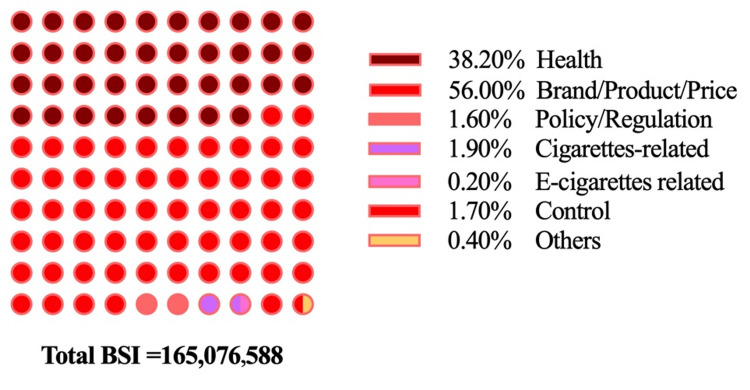
The theme categories of e-cigarette related search in the Baidu index user demand module.

The user's demands and concerns were reflected in the retrieved search words by the Baidu index platform. From the user demand module, the user’s main concerns and detailed inquiries for each search keyword were manifested by these related search keywords. We categorized those retrieved related words into 10 themes based on their content: 1) e-cigarettes-related, 2) cigarettes-related, 3) medication; 4) policy and regulations; 5) control; 6) health; 7) others; 8) brand/product/price; 9) biology, and 10) irrelevant. Irrelevant inquiries were evaluated by three independent investigators and were agreed to be excluded from this study. Among the remaining nine themes, biology and medication were not found to be increasing in frequency. The summed valid BSI of e-cigarette related words was 165,076,588. The quality of e-cigarettes and their health-related problems concern the public the most. E-cigarette brand, product, and price-related inquiries were in the lead among all the inquiries, with a BSI that exceeds the sum of all the other themes combined. Aside from these, there were 1.90% cigarette-related inquiries, 1.60% policy and regulation related inquiries, 1.70% control inquiries, 0.20% e-cigarette related inquiries, and 0.4% other inquiries. The health-related inquiry with 38.20% was ranked second (Figure 6).

## Discussion

The principal findings of our study are that e-cigarette policy and regulation lag behind its rapid market extension. Individuals who were 19 years of age or younger expressed a strong desire to acquire additional knowledge regarding e-cigarettes. Notably, the number of inquiries made by females exceeded that of males by approximately 1.5 times. From January 1, 2011, to April 4, 2022, the definition of e-cigarettes was the most frequently searched keyword, followed by "Hazards from E-Cig" and "Hazards caused by E-Cig," which can be regarded as health concerns. We found that searches related to definitions, health issues, and product and promotion themes initially experienced slow growth in the BSI. However, they have been rising steadily since 2014, with the first peak appearing on April 15, 2015. These searches then dramatically increased in July 2020 and remained high for the last three years. We can infer that the reason for these results may be attributed to the fact that the promotion and sale strategy of e-cigarettes was primarily online and that its market was experiencing rapid growth in 2014. It also coincides with the fact that e-cigarette manufacturing was upsizing in 2014 and posed a great challenge to the traditional tobacco cigarette market and may eclipse tobacco cigarettes eventually [35,36]. Among those three themes, the trends of product and promotion were relatively low and significantly separated from the other two themes. However, the BSI of policy and regulation themes was generated in 2021; therefore, the AAPC cannot be calculated based on less than two years of searching data. It also reflected that e-cigarettes were relatively underregulated in its growing market, and the surveillance of e-cigarette marketing campaigns, promotions, and target customers was not ensured and well protected [13].

As for the geographical regional distribution differences, we found that approximately 50% of search records were from the north and east regions, and only 16% were from both the northwest and northeast regions combined. These findings could be potentially attributed to the variations in the population distribution and the economic makeup. The population in China is unevenly distributed, with approximately 94% of the population concentrated in only 43% of the land area. This concentration of population in the southeast region has resulted in high population density, while the northwest region has a lower population density [37]. The socioeconomic status of the eastern coastal region is higher than the central and western regions. People living in the early urbanized eastern coastal cities have better public health awareness and more information access from various resources, and e-cigarettes may provide an option for them to express individuality and a healthy alternative to tobacco [38]. In line with gender norms, it is generally anticipated that there would be a male predominance in the inquiries. However, in our study, the inquiry volume from the female gender was approximately 1.5 times as many as the male gender. It may reveal different concerns and interests between males and females. In the context of the increasing incidence of lung cancer morbidity in females, especially in non-smokers, it is evident that females are more prone to engage in online health information seeking behavior with a specific focus on the etiology of lung cancer [17].

Meanwhile, the age distribution of the search inquiries aligns with other studies, indicating that young and middle-aged people had the most inquiry volumes. According to our study, 11.59% of search volumes were from those aged 19 years and younger. A previous study by Chen et al. [39] also revealed that tobacco use was found in adolescents aged 12-18 years, with some of them with even dual use of e-cigarettes and traditional cigarettes. Search volume from those who were over 50 years of age only accounted for 4.8%, as they may be more inclined toward traditional tobacco cigarette smoking. It is posited that the youth demographic exhibits a sense of curiosity and that they seem to embrace novelty. The variety of flavors and attractive designs of e-cigarettes have expanded the consumer base. Moreover, during the initial stages, aggressive promotions and marketing channels of e-cigarettes were predominantly online, which was hard to regulate and even provided minors with access to them [10]. Middle-aged people are more receptive to the narrative behind the promotion of e-cigarettes, which is supposedly healthier and more socially acceptable [3]. Although e-cigarettes were invented quite ideally initially, with no tar and no carcinogens, the so-called “harmless and healthier” advantages may encourage more younger people to experiment with them and increase the likelihood of developing a smoking habit [21]. Moreover, the general public’s understanding of the adverse effects caused by e-cigarettes remains limited [40]. Hence, the use of e-cigarettes is becoming a concern for both health professionals and policymakers, and it needs prompt regulation and laws to supervise the sales market and standardize the manufacture strictly and to protect and guide the public in the right direction. In China, in April 2022, the State Administration for Market Regulation (the State Standardization Administration) announced the approval and promulgation of mandatory national standards for e-cigarettes [41], with effect from October 1, 2022. The national standard for e-cigarettes (GB 41700-2022) has been implemented. This standard specifies product specifications, models, and parameters aimed at discouraging minors from using e-cigarettes and preventing children from initiating their use [42].

From the user demand domain, the result revealed that those public concerns are mainly e-cigarette product quality and health-related problems. However, the health damage of e-cigarettes and public perception are disputable with their increasing popularity. Based on the search keywords "How much damage could be caused by E-Cig," "Alterations in Lungs for 1-year E-cig," "Is E-Cig hazardous?" and "E-Cig or cigarette, which is more hazardous?" it can be inferred that public concerns about the hazards of e-cigarettes persist, despite limited studies asserting that e-cigarettes have no adverse health effects and can aid in quitting smoking [43]. Vardavas et al.'s research shows that e-cigarette smoking induces immediate adverse physiologic effects, which are similar to some of the effects seen with tobacco smoking [44,45], or even worse outcomes, including unexpected consequences such as lung injury, eosinophilic pneumonitis, exogenous lipoid pneumonia, and inflammatory reaction mimicking metastatic cancer [36,46]. E-cigarettes are not free of nicotine or carcinogens, and the consequences of nicotine addiction, dual use of traditional combusted cigarettes and e-cigarettes, and smoking exposure persist. Additionally, people might inhale more nicotine and carcinogens due to propylene glycol and/or vegetable glycerin being added to vape liquid, which brings a better taste, sense, and experience for smokers [47]. Our study has found that the search volume from individuals aged 19 years and below reached almost 12%, which aligns with broader research that demonstrates concerns about minors using e-cigarettes [41,48]. The study also highlighted a scenario in which adolescent boys using e-cigarettes containing tetrahydrocannabinol experienced lung injury outbreaks [49].

To date, smoking remains a risk factor for many types of cancers, including breast cancer, bladder cancer, prostate cancer, cervical cancer, and lung cancer [12,50]. The prevalence of the smoking habit not only increases the number of smoking-related diseases but also contributes to the socio-economic burden [51]. As for lung cancer, e-cigarettes are also a potential risk factor. The toxic chemicals and carcinogens in vaping liquids may injure the upper respiratory tract and lungs directly, and the cancerogenic substances may induce inflammatory reactions, immune responses, and carcinogenesis [47]. Given the escalating concerns in e-cigarette consumption, it is essential to conduct investigations to explicate the long-term implications of e-cigarette exposure in both adolescents and adults. Our study profiles the public concerns regarding e-cigarettes, providing a new perspective for evaluating e-cigarettes. Our analysis, which incorporates trends in online search behavior, geodemographic factors, and search demand patterns, provides a comprehensive examination of information from various dimensions. These findings can serve as a valuable resource for health experts, offering insights to guide further investigation and inform decision-making processes. Furthermore, it is worth noting that searches related to regulation and medication were sparse. The regulation and standards of e-cigarettes should be strictly implemented and supervised while avoiding persuasive advertising campaigns and illicit sales.

Limitations

There are several limitations of this study that need to be addressed. Firstly, the search engine, Baidu, used for this study is a dominated search engine in a certain region, and the recorded data are from its registered users’ search. Due to discrepancies in how users search for information about the topic, it can be challenging to calculate their demand directly. However, by analyzing the relevant search terms and identifying common patterns, we can gain insight into their interests and preferences. Secondly, the platform only displayed the data for the last decade, and thus the earlier trend could not be analyzed. Thirdly, the data recorded on the Baidu Index platform are limited to search keywords that meet an established threshold for user access. Therefore, the low use or unusual expressions may not be included in the trend analysis. However, the platform offers a valuable source of daily updated data for future investigations and real-time analysis.

## Conclusions

E-cigarettes enjoy great popularity nationwide, but product quality and safety are major public concerns. Regulation of e-cigarettes for their standard production, quality control, advertisement, and target customers should be implemented promptly, and the public needs to have a clear perception of e-cigarettes, especially adolescents. E-cigarette related health damages or consequences require further investigation, and advertisements and promotions for e-cigarettes should be strictly controlled by the government.
